# Characterization of Epiphytic Bacterial Communities from Grapes, Leaves, Bark and Soil of Grapevine Plants Grown, and Their Relations

**DOI:** 10.1371/journal.pone.0073013

**Published:** 2013-08-30

**Authors:** Guilherme Martins, Béatrice Lauga, Cécile Miot-Sertier, Anne Mercier, Aline Lonvaud, Marie-Louise Soulas, Guy Soulas, Isabelle Masneuf-Pomarède

**Affiliations:** 1 USC Oenologie-INRA, Université Bordeaux Segalen, ISVV, Villenave d'Ornon, France; 2 Bordeaux Science Agro, Gradignan cedex, Bordeaux, France; 3 Equipe Environnement et Microbiologie UMR IPREM 5254 IBEAS, Université de Pau et des Pays de l'Adour, Pau, France; Agricultural Research Service, United States of America

## Abstract

Despite its importance in plant health and crop quality, the diversity of epiphytic bacteria on grape berries and other plant parts, like leaves and bark, remains poorly described, as does the role of telluric bacteria in plant colonization. In this study, we compare the bacterial community size and structure in vineyard soils, as well as on grapevine bark, leaves and berries. Analyses of culturable bacteria revealed differences in the size and structure of the populations in each ecosystem. The highest bacteria population counts and the greatest diversity of genera were found in soil samples, followed by bark, grapes and leaves. The identification of isolates revealed that some genera – *Pseudomonas, Curtobacterium*, and *Bacillus* – were present in all ecosystems, but in different amounts, while others were ecosystem-specific. About 50% of the genera were common to soil and bark, but absent from leaves and grapes. The opposite was also observed: grape and leaf samples presented 50% of genera in common that were absent from trunk and soil. The bacterial community structure analyzed by T-RFLP indicated similarities between the profiles of leaves and grapes, on the one hand, and bark and soil, on the other, reflecting the number of shared T-RFs. The results suggest an interaction between telluric bacterial communities and the epiphytic bacteria present on the different grapevine parts.

## Introduction

Plants sustain a complex microecosystem, which harbours a diverse array of bacteria, able to colonize different plant organs and tissues, including roots, leaves, flower clusters, seeds and fruits [1], [2]. In so doing, plant-associated bacteria can affect crop health, due to their capacity to suppress or stimulate the colonization of tissues by plant pathogens [3]. In recent years, these bacteria have attracted considerable academic attention for their potential biotechnological applications [4], [5].

So far studies of bacteria associated with grapevines have mainly focused on pathogens responsible for plant diseases, like Pierce's – caused by *Xylella fastidiosa* subsp. *fastidiosa* [6]; Crown gall – caused by *Agrobacterium tumefaciens* [7]; and bacterial blight, caused by *Xylophilus ampelinus* [8]. More recent investigations have studied the diversity of the grapevine endophytic bacteria, and have shown that *Pseudomonas* and *Bacillus* sp., can act as biological disease suppression agents, stimulating plant growth and health [9], [10], . Few studies have investigated epiphytic bacteria on grapevines. Most research has concentrated on bacteria of oenological interest, like acetic and lactic acid bacteria [12], [13], [14], [15], [16], present in the microflora on grape berries. Little information is available about the effects of epiphytic bacteria on other plant parts, like leaves and bark. In a recent study, using high-throughput sequence analysis of 16s rRNA, Leveau and Tech [17], showed that the bacterial community on leaves differed, both in size and structure, from that on berries. They also reported a large diversity of bacteria associated with leaves and grapes, belonging to species known to be plant growth-promoters with significant activity against grapevine pathogens.

Therefore, grapevine bacteria play a key role not only in plant health, but also in crop quality and yields. The surface of grape berries represents a natural reservoir of bacterial microbiota that has various impacts on the sanitary quality of grapes and may influence the winemaking process, with major repercussions on wine quality [14], [16], [18]. Despite their importance, the diversity of epiphytic bacteria on grape berries remains poorly described, as is the role of other plant parts and vineyard soil in bacterial colonization. Soil microorganisms are able to colonize parts of the plant above the ground, including leaves and fruit [19], [20], [21]. Previous studies revealed the presence of pathogenic fungi and peronosporomycete, such as *Erysiphe necator* (formerly *Uncinula necator*) and *Plasmopara viticola* in vineyard soil [22], [23], which also affect aerial parts of plants, like leaves, stems, flowers, and fruit.

Oenologically-important microorganisms, like *Saccharomyces cerevisiae*, the main yeast responsible for alcoholic fermentation, have been also isolated from vineyard soil [24], [25], [26].

Few studies have reported the presence in soil of bacterial species associated with grape berries. For instance, some lactic and acetic acid bacteria species found in wine environments have also been detected in vineyard soil [27].

These results suggest that vineyard soil may be a source of primary inoculum, able to participate in the structure of the microbial community on the aerial parts of the vine, including grapes. However, no comparative studies of the structure of bacterial communities on grapevine parts and in soil had previously been conducted. Consequently, it is difficult to evaluate the potential of plant- and soil-associated microflora to colonize grape berries.

In this study, culture-dependent and -independent methods were used to characterize and compare the bacterial community size and structure in vineyard soil, and on grapevine bark, leaves, and berries. Specific taxa from each ecosystem as well as taxa common to all ecosystems were identified. The results provide insights into the relations between epiphytic bacteria in different vineyard ecosystems.

## Materials and Methods

### Site description and sampling design

This study was performed in the Lussac St. Emilion winegrowing region, southwest France (44°57′15″N 0°06′12″ W, 77 m altitude), in 2010. Two vineyards were selected, spaced 400 meters apart. Both vineyards had very similar characteristics: grape variety (Merlot), age, pruning system, canopy management, and sun exposure.

Samples were collected at the beginning of berry ripening, corresponding to stage 34 of the modified E-L system for identifying major and intermediate grape vine growth stages [28]. Three sampling points were selected in each vineyard, each corresponding to five vines.

### Ethics statement

All the samples in this study were collected on private proprieties, and the owners of the vineyards gave permission to conduct the study on these sites. No specific permissions were required for these locations, because there are no endangered or protected species in these areas, and this study did not involve endangered or protected species.

### Soil sampling

Soil samples were collected in five randomly-chosen plots around each vine selected (15 to 25 cm from the trunk) at Ap horizon (0–5 cm depth) and mixed in sterile plastic bags. Samples were transported to the laboratory in refrigerated boxes and analyzed within 12 h after collection. Fresh soils were sieved (Ø<2 mm) to remove plant residues, soil macrofauna and stones. The soils had the following physico-chemical characteristics: 276 g/kg clay, 467 g/kg silt, 80 g/kg fine sand; 176 g/kg coarse sand; 39.8 g/kg OM, C/N 12, pH 6.59, CEC 13.1 cmol+/kg; in vineyard *I* and 354 g/kg clay, 373 g/kg silt, 80 g/kg fine sand; 189 g/kg coarse sand; 36 g/kg OM, C/N 11.5, pH 7.54, CEC 17.7 cmol+/kg in vineyard *II*.

### Sampling grape berries, leaves and bark

At each sampling point, approximately 1 kg healthy, undamaged grapes, with their pedicels attached, was aseptically removed from several bunches and placed in sterile bags. Leaf and bark samples were collected from the same vines. Bark was collected avoiding damage to living tissue. To prevent cross contamination, sampling tools were sterilized with 75% ethanol before each sample. All samples were transported to the laboratory in refrigerated boxes and processed within 12 h.

### Microbial biomass recovery

From each soil sample, aliquots of 1 g (dry weight equivalent) were taken, dispersed in a solution (10 ml) containing sodium hexametaphosphate (35 g/l) and sodium carbonate (7 g/l), and subjected to orbital shaking (Vibrax VXR Basic, 1200 rpm) for 1 h. These cell suspensions were used for downstream analysis.

Grape berry samples: 250 undamaged berries were randomly, aseptically removed from the bunches, placed in sterilized flasks with 500 ml isotonic solution containing 0.1% peptone and 0.01% Tween 80, and subjected to orbital shaking at 150 rpm for 1 h [29]. Leaves and bark: 50 g samples of each were placed in sterilized flasks and washed with 250 ml of the isotonic solution described above. These cell suspensions were used for downstream analysis. One part of the suspension was used to inoculate the culture medium and the rest was filtered through a 0.2 µm pore size, 47-mm diameter cellulose acetate filter (Sartorius AG, Göttingen, Germany) held in a stainless steel vacuum filtration unit (Millipore, Hertfordshire, UK).

### DNA extraction

Soil DNA was extracted from 1 g (dry weight equivalent) soil using the UltraClean soil DNA isolation kit (MoBio Inc., Solana, CA, USA), as described by the manufacturer. DNA was extracted directly from the microbial biomass of grape berries, bark, and leaves retained on the membranes used to filter the cell suspensions.

The membranes were aseptically cut into small pieces and placed on the bead solution tubes provided by UltraClean soil DNA isolation kit. The extraction protocol was then continued, as described by the manufacturer.

### Colony isolation and counting

Aerobic and aero-tolerant bacteria from the cell suspensions extracted from the soil, grape berries, leaves, and bark were cultured after spreading tenfold serial dilutions on 1/10 diluted LB culture medium (1 g/l bactotryptone, 1 g/l yeast extract, 0.5 g/l NaCl, and 20 g/l agar) with 150 mg/l biphenyl (Acros Organics, Belgium) to inhibit yeast and mould growth. Each dilution was prepared in triplicate.

Plates were incubated under aerobic conditions at 25°C. The number of colony-forming units was counted 5 days after inoculation. From each ecosystem in both vineyards, around 30 colonies were randomly picked from 1/10 diluted LB medium and purified by streaking onto fresh 1/10 diluted LB plates. The purity of each colony was verified and they were stored at −80°C on 33% glycerol stock for further genetic identification.

### Identifying isolates by their 16 S rRNA gene sequence

The DNA was extracted from the isolates and stored using the FTA® CloneSaver™ card (Whatman® BioScience, USA), as described by Zott [30]. DNA was used as template for PCR amplification with 16S rRNA primers 8F (5′-AGAGTTTGATCCTGGCTCAG-3′) and 1063R (5′-ACGGGCGGTGTGTRC-3′) [31]. After Sanger sequencing of the amplicons (Plateforme Génomique Fonctionnelle, Université Victor Segalen, Bordeaux 2, France), the sequences were aligned and compared with references in the GenBank, using the NCBI Basic Local Alignment Search Tools BLASTn program (http://www.ncbi.nlm.nih.gov/BLAST). The identification was considered valid when the identity of a contiguous sequence of 343 bp-989 bp was at least 98%. The 16 S rDNA sequences obtained were deposited in the EMBL Nucleotide Sequence Database under accession numbers HF566150 to F566373.

### T-RFLP analysis of bacterial communities

DNA extracted from cell suspensions obtained from the soil, grape berries, leaves, and bark was amplified using nested PCR. A 1398 bp region of the 16S rRNA gene was first amplified using primers 8F and 1406R. In the second step, a 1055 bp region was amplified using primer 8F, fluorescently labelled at the 5′ end with 6-FAM (6-carboxyfluorescein), and 1063R (5′-CTCACGRCACGAGCTGACG-3′) [29]. The PCR was run in a final volume of 50 µl containing 10 mM Tris-HCl, 50 mM KCl, 1.5 mM MgCl_2_, 0.2 mM dNTPs, 5% glycerol, 0.08% NP-40, 0.05% Tween-20, 25 units/ml Taq DNA polymerase, and 200 nM of each primer. PCR conditions for the first amplification step were: 95°C for 5 min, 20 cycles at 94°C and 58°C for 1 min each, 72°C for 1.5 min, and 72°C for 7 min. The same conditions were used for the second amplification step, reducing the number of cycles to 15. PCR products (100 ng) were purified using the Geneclean Turbo Kit (Qbiogene) and digested with 3 U *Hae* III or *Hinf* I enzymes (New England Biolabs). Fluorescently-labelled terminal-restriction fragments (T-RFs) were separated by capillary electrophoresis on an ABI prism 310 (Applied Biosystems). About 10 ng digested DNA was mixed with 9 μl de-ionised formamide and 0.5 μl 5-carboxytetramethylrhodamine size-standard TAMRA 500 (GeneScan™), denatured at 95°C for 5 min, and immediately chilled on ice prior to electrophoresis. After a 10 s injection step, electrophoresis was performed at a voltage of 15 kV for 30 min. T-RFLP peaks were analysed using GeneScan software (ABI), profiles were compiled to produce data matrices, background noise was reduced, and T-RF heights were normalized [32]. Only T-RFs between 50 bp and 500 bp were analysed. The profiles were aligned using T-align software [33], with a confidence interval of 1.0.

The 16 S rRNA gene of vine chloroplast was analyzed in order to eliminate operational taxonomic units (OTUs) of plant DNA. DNA was extracted from *in vitro*-grown merlot vine plantlets and used as a PCR template. PCR amplification and enzyme restriction with *Hin*fI and *Hae* III was carried out following the protocol described in 2.6. The profile generated revealed one peak with a molecular weight of 178 bp (*Hinf* I) and one with a molecular weight of 295 bp (*Hae* III). These two T-RFs were excluded from the sample profiles.

### Statistical data analyses

#### Culture data

The statistical significance of the differences between bacterial counts in samples from the different ecosystems was tested by one way ANOVA followed by Tukey's honestly significant difference test (Tukey's HSD, p<0.001).

#### T-RFLP analysis

Statistical data were analyzed using Statistica V.7 software (Statsoft Inc., Tulsa, OK, USA). The phylotype richness (*S*) was calculated as the total number of distinct T-RF peaks in each normalized profile. Shannon–Weaver diversity indices *(H'* *)* were calculated using peak heights as a metric of abundance for each T-RFLP profile, as described previously [34].
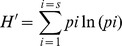



Shannon–Weaver evenness indices (*E*) were calculated as *H'*/*H'*max, where *H'*max  =  ln *(S)*. Principal component analysis of T-RFLP profiles was performed with samples as statistical observations and T-RFs as variables. Profiles were plotted as dots in the factor space and the original variables (each T-RF) were represented by arrows. The direction and length of the arrows indicate the contribution of the variables along the principal components.

To evaluate similarities between T-RFLP profiles, hierarchical clustering analysis was performed based on binary data, presence or absence of T-RFs, for all individuals. The Jaccard's index was used to quantify the similarity between datasets and UPGMA (Unweighted Pair Group *Method with* Arithmetic Mean) as a clustering method., The statistical significance of the differences between Shannon–Weaver diversity (H') and Evenness indices (E) from 16 S rRNA gene T-RFLP profiles was tested by one way ANOVA followed by Tukey's HSD test, (p<0.001).

## Results

### Size of culturable bacterial communities from the different plant parts and soil

Table 1 shows the culturable bacteria population densities. In both vineyards, the highest bacteria population counts were detected in the soil samples. The next highest was found in bark samples, followed by grapes and leaves. Tukey's honestly significant difference test (Tukey's HSD) was performed post-hoc. The results showed five homogeneous groups: soil from vineyards, bark from both vineyards, grapes from both vineyards, leaves from vineyards *I* and leaves from vineyard *II* respectively.

**Table 1:** Culturable bacteria populations in samples of soil, bark, leaves, and grape berries.

Samples	log _10_ CFU/g of fresh weight,	Tukey grouping				
		1	2	3	4	5
Leaves (vineyard I)	3,49 (±0.11)				****	
Leaves vineyard II	3,92 (±0.12)					****
Grapes (vineyard I)	4,52 (±0.04)	****				
Grapes vineyard II	4,65 (±0.09)	****				
Bark vineyard II	5,02 (±0.11)		****			
Bark (vineyard I)	5,15 (±0.12)		****			
Soil vineyard II	6,88 (±0.08)			****		
Soil (vineyard I)	6,97 (±0.08)			****		

Results expressed as log _10_ CFU/g of fresh weight, determined by plate counting on LB 1/10 medium. The values are the average of a triplicate experiment ± standard deviation. * denotes homogenous groups revealed by post-hoc tests (Tukey's HSD multiple-comparison test, p<0.001).

### Diversity of culturable bacteria within different samples

From the randomly picked colonies, 5% and 7% did not survive subculturing, from vineyards *I* and *II*, respectively. From 16S rDNA sequence analysis, the 224 remaining isolates were assigned to a specific genus, with a classification threshold above 98% (Table 2). The distribution of each genus varied according to the type of sample. However, there were similarities in the diversity and abundance of isolates from each ecosystem in both vineyards.

**Table 2:** Diversity of culturable bacteria within different samples.

Bacterial genera	Source of isolation				Source of isolation				
	Vineyard I				Vineyard II				
	Grapes	Leaves	Bark	Soil	Grapes	Leaves	Bark	Soil	Total
***Acinetobacter sp.***	0	0	0	1	0	0	0	0	**1**
***Agrobacterium tumefaciens***	0	0	1	1	0	0	1	1	**4**
***Bacillus sp.***	2	3	2	3	2	4	3	4	**23**
***Brevibacterium sp.***	0	0	1	0	1	0	1	0	**3**
***Burkholderia sp.***	1	0	2	1	1	0	0	1	**6**
***Cellulomonas sp.***	1	0	4	1	1	0	2	1	**10**
***Clostridium sp.***	0	0	0	5	0	0	0	4	**9**
***Curtobacterium sp.***	1	6	3	2	1	4	3	4	**24**
***Enterobacter sp.***	2	0	0	1	3	0	0	2	**8**
***Massilia sp.***	4	2	0	0	3	1	0	0	**10**
***Micrococcus sp.***	2	0	1	1	3	0	1	3	**11**
***Paenibacillus sp.***	0	0	0	0	0	0	0	2	**2**
***Pantoea sp.***	0	0	2	1	0	0	2	1	**6**
***Pseudomonas sp.***	9	10	5	3	7	10	4	3	**51**
***Rhizobium sp.***	0	0	1	2	0	0	0	2	**5**
***Sphingomonas sp.***	1	8	0	1	3	7	0	1	**21**
***Staphylococcus sp.***	0	0	2	1	0	0	2	1	**6**
***Streptococcus sp.***	0	0	0	1	0	0	0	0	**1**
***Streptomyces sp.***	0	0	1	1	0	0	1	1	**4**
***Xanthobacter sp***	0	0	2	1	0	0	3	1	**7**
***Xanthomonas sp.***	0	2	3	0	0	1	2	0	**8**
***Xylanimonas sp.***	0	0	1	0	0	0	0	0	**1**
***Xylaphtlus sp.***	0	0	1	0	0	1	0	0	**2**
***Xylella sp.***	0	0	1	0	0	0	0	0	**1**
**Total**	**23**	**31**	**33**	**27**	**25**	**28**	**25**	**32**	**224**

A total of 24 different genera were identified. The strains belonged to 6 different bacterial classes (*alpha-, beta-, and gamma-Proteobacteria, Actinobacteria, Clostridia,* and *Bacilli*).

Soil samples contained 16 and 17 genera, in vineyard *I* and *II,* respectively, followed by bark (14 and 12), grapes (9 and 10), and leaves (5 and 6). In grape samples, the genus *Pseudomonas* was the most abundant, followed by *Massilia, Micrococcus,* and *Bacillus,* but - unlike leaves, bark and soil – the occurrence of *Curtobacterium* sp. was low. In leaves, *Sphingomonas* and *Pseudomonas* were the most abundant genera, followed by *Bacillus* and *Curtobacterium*. Bark samples contained the highest percentage of *Xanthobacter*, *Xanthomona*s, and *Cellulomonas*. In soil samples, the predominant genus was *Clostridium*, followed by *Bacillus* and *Rhizobium.*

Some of the genera – *Pseudomonas*, *Curtobacterium* and *Bacillus –* were present in all ecosystems, although with varying abundance. Some other genera were ecosystem-specific, such as *Xylella* and *Xylanimonas* in bark and *Acinetobacter, Clostridium, Streptococcus*, and *Paenibacillus* in soil. Grape and leaf ecosystems did not contain any specific genera, but *Massilia* spp. was present in both grapes and leaves and absent from the other samples (soil and bark). Some genera (*Staphylococcus, Streptomyces, Rhizobium, Agrobacterium, Xanthobacter, Pantoea)* were always found together in soil and bark, but were absent from leaves and grapes. *Brevibacterium* were present in grapes and bark and *Enterobacter and Burkholderia sp*.in grapes and soil. Xylophilus was only present in leaves and bark. Between 42 and 50% (vineyard *I* and *II*, respectively) of bacterial genera identified in grape samples were also found on leaves. Around 55% of the genera identified in soil samples were also found in bark.

Taking into account some culture bias for the growth of specific microorganisms associated with culture-based detection method, we decided to use T-RFLP as community profiling tool to study the bacterial community structure from grapes, leaves, bark and soil.

### Bacterial community structure analyzed by T-RFLP

Although the internal size marker ranged from 35 to 500 bp, no major T-RF peaks were found below 59 bp or above 492 bp. The samples showed considerable variability in the number of T-RFs and fluorescence intensity. In the profiles generated by *Hinf* I and *Hae* III digestion, a total of 69 and 72 different T-RFs, respectively, were conserved for analysis. Although there was only a slight difference in the number of unique T-RF peaks obtained using the two enzymes, the soil sample profiles obtained with *Hae* III generally exhibited more T-RF peaks than those obtained with *Hinf* I. As to the evenness and diversity of the different profiles, there was a significant difference in terms of the (E) and (H') index (Table 3). Soil samples presented the highest (E) and (H') values, whereas leaves presented the lowest. Post-hoc tests revealed different homogeneous groups (Tukey's HSD, P<0.001). In both vineyards, the evenness and diversity values obtained with *Hinf* I revealed two groups: leaves and grapes on the one hand, and bark and soil on the other. The corresponding values obtained with *Hae* III fell into three different groups. Leaves and grapes exhibited the least diversity and evenness; bark had an intermediate degree; and soil had the highest degree of both. A Pearson correlation analysis revealed a positive correlation between (E) and (H') values (r = 0 .79, p-value <0.001).

**Table 3:** Shannon–Weaver diversity and Evenness indices from 16S rRNA gene T-RFLP profiles.

A Vineyard *I*	Evenness (E)	Tukey grouping			Diversity (H’)	Tukey grouping		
*(Hinf* I*)*		1	2			1	2	
Leaves	0.57 (+/−0.08)	**			1.58 (+/−0.30)	**		
Grape berries	0.59 (+/−0.04)	**			1.56 (+/−0.26)	**		
Soil	0.68 (+/−0.04)		**		2.28 (+/−0.19)		**	
Bark	0.82 (+/−0.09)		**		2.68 (+/−0.1)		**	
(***Hae*** ** III**)		1	2	3		1	2	3
Leaves	0.42 (+/−0.09)	**			1.16 (+/−0.26)	**		
Grape berries	0.5 (+/−0.02)	**			1.33 (+/−0.36)	**		
Soil	0.89 (+/−0.03)		**		3.21 (+/−0.02)		**	
Bark	0.79 (+/−0.04)			**	2.68 (+/−0.11)			**

B Vineyard *II*	Evenness (E)	Tukey grouping			Diversity (H’)	Tukey grouping		
*(Hinf* I*)*		1	2			1	2	
Leaves	0.65 (+/−0.08)	**			1.89 (+/−0.15)	**		
Grape berries	0.43 (+/−0.09)	**			1.22 (+/−0.13)	**		
Soil	0.75 (+/−0.05)		**		2.44 (+/−0.1)		**	
Bark	0.7 (+/−0.02)		**		2.26 (+/−0.2)		**	
(***Hae*** ** III**)		1	2	3		1	2	3
Leaves	0.66 (+/−0.06)	**			1.94 (+/−0.28)	**		
Grape berries	0.6 (+/−0.03)	**			1.71 (+/−0.07)	**		
Soil	0.88 (+/−0.02)		**		3.17 (+/−0.02)		**	
Bark	0.83 (+/−0.03)			**	2.74 (+/−0.1)			**

Values in brackets represent standard deviation (n = 3). * denotes homogenous groups revealed by post-hoc tests (Tukey's HSD multiple-comparison test, p <0.001) based on the comparisons between the (E) and (H') values of different samples (n = 6).

### Comparison of bacterial colonization patterns in different ecosystems

**Figure 1:**
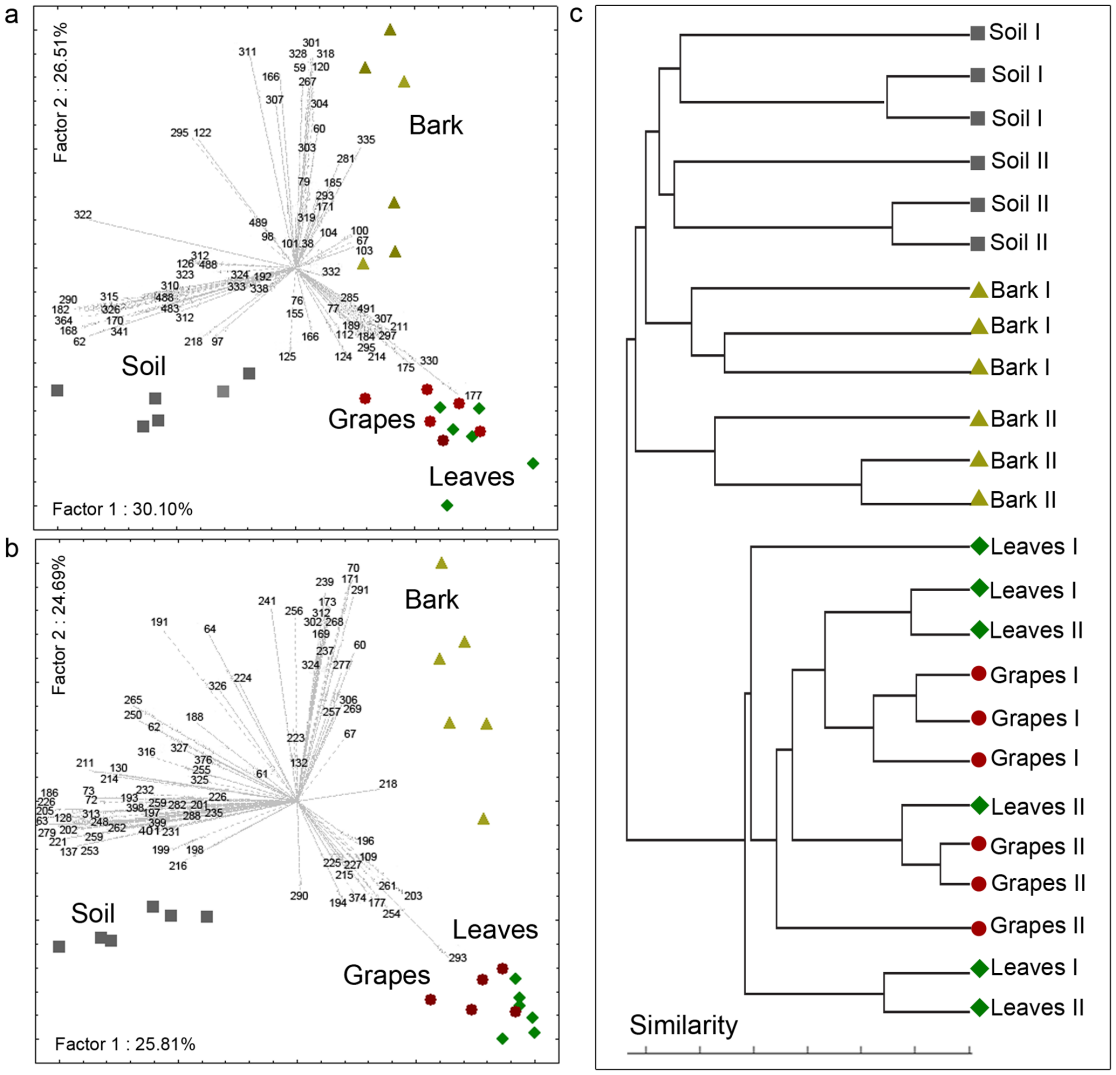
Comparison of bacterial colonization patterns in different ecosystems. Principal-component analysis based on bacterial community structure, assessed by 16S rRNA gene T-RFLP (including TRF size and relative abundance data), using Hinf I digest, from vineyard I (a) and vineyard *II* (b); the amount of variability accounted for by each factor is shown on the axes. (c) Clustering obtained by the hierarchical UPGMA method based on the arithmetical complement of the Jaccard similarity index of samples from both vineyards. Triangles (Δ) indicate bark samples; (◊) diamonds indicate leaf samples, squares (□) indicate soil samples; circles (○) indicate grape samples.

**Figure 2:**
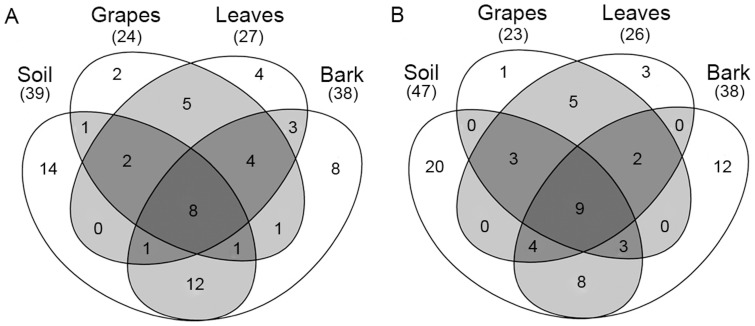
Four-way Venn diagram depicting the shared and unshared T-RFs between the four niches. Profiles obtained with *Hinf* I (A) and *Hae* III (B) endonucleases from samples of both vineyards *I* and *II*. Numbers in parenthesis indicate the total number of TRFs for each niche.

The results of PCA analysis of the T-RFLP profiles of the samples from each vineyard are shown in Figure 1 a and b. The samples were represented as four different types of dots, according to the Figures 2a and 2b represent the profiles obtained with *Hinf* I for samples from vineyards *I* and *II*. PCA demonstrated that the profiles obtained from leaves and grapes were very similar to each other. Samples from both vineyards were grouped together on the plot, indicating similarities between the bacterial communities present in these two ecosystems. Soil samples were clustered together on the opposite side of the plot from bark. In both vineyards *I* and *II* soil and bark samples were clearly distinguishable from those from leaves and grapes.

In order to assess the impact of the vineyard on the profiles, all the samples together were subjected to cluster analysis. Based on the profiles obtained using the *Hinf* I enzyme (Figure 1c), two major groups were defined – soil and bark versus leaves and grapes. In the soil and in bark sub-groups, there was a clear difference between vineyards *I* and *II*. Leaves and grapes were grouped together in several clusters, with no significant inter-vineyard differences. The only exception was grape samples from vineyard *I*, which formed a separate cluster. The results obtained using *Hae* III digestion were similar (data not shown).

The similarities between the leaf and grape profiles, on the one hand, and bark and soil, on the other, reflect the number of T-RFs shared by those samples. Soil and bark samples shared a large number of T-RFs, as did leaves and grapes. In contrast, neither soil nor bark shared many T-RFs with either leaves or grapes. A 4-way Venn diagram (Figure 2) of T-RFs revealed that soil and bark had more exclusive T-RFs than either leaves or grapes. Some T-RFs were present in all ecosystems; 8 and 9 T-RFs with *Hinf* I and *Hae* III digestion, respectively, were common to all profiles.

### Phylogenetic assignment of T-RFs

PCA revealed correlations between T-RFs and each ecosystem. The T-RFs with larger factor loading in the direction of each cluster were selected. Their putative identities were predicted by *in silico* digestion with both *Hinf* I and *Hae* III enzymes, using the Phylogenetic Assignment Tool (PAT+) [35] provided by Microbial Community Analysis III (MiCA 3) (http://mica.ibest.uidaho.edu) [36], based on the RDP Release 10, 16S rRNA gene data base. One limitation of this kind of analysis is the inability to affiliate OTUs to phylogenetic groups with any degree of reliability, since T-RFs of the same size may yield phylogenetically disparate 16 S rRNA gene sequences [37]. In order to minimize this bias and obtain more reliable identification, profiles obtain with both enzymes were analyzed. Phylogenetic assignment of the T-RFs was grouped at the phylum level. Members of *Actinobacteria*, *Firmicutes,* and *Proteobacteria* corresponded to T-RFs present in all different clusters. The soil clusters were dominated by species classified as *Actinobacteria*, *Betaproteobacteria,* and *Clostridia*. Bark clusters presented T-RFs assigned to *Actinobacteria* and *Clostridia* and a considerable number of *Proteobacteria* (*Alphaproteobacteria, Epsilonproteobacteria,* and *Gammaproteobacteria*). The majority of T-RFs from leaves and berries were classified as *Proteobacteria* (*Gammaproteobacteria* and *Alphaproteobacteria*).

## Discussion

Recent studies indicate that bacterial populations on wine grapes are much more diverse than previously supposed [38], [18]. Little is known about the diversity of the epiphytic bacteria associated with other grapevine parts, such as leaves or trunk bark, as well as bacteria living in vineyard soil. In this work, culture-dependent and independent methods were combined to compare the composition of epiphytic bacterial communities present on different plant parts and in soil. These approaches revealed differences in population density and diversity among different samples from the various ecosystems. Soil and bark hosted a greater diversity and species richness than grapes and leaves.

In addition, a comparison of all the profiles from both vineyards revealed similarities between leaves and grapes, on the one hand, and bark and soil, on the other.

Many factors are likely to be involved in determining the species composition of bacterial communities in plants and soil. They include the availability of immigrant inoculum [39], [40], host plant phenology [41], [38], physico-chemical environmental conditions [42], [43], and nutritional characteristics of the phyllosphere or soil [4], [44]. The variability in nutrient supply between these niches may partly explain the differences in bacterial community structure observed, as well as the diversity of culturable genera among the different plant parts and in soil.

Soil provides a large variety of carbon sources, including amino acids, organic acids, and carbohydrates, used by micro-organisms to obtain energy [45]. Bark contains starch, sugars [46], [47], and other nutrients from xylem sap exudation [48]. These two nutrient-rich ecosystems are favourable to a greater number and more diverse range of bacteria.

The fact that bark contains high concentrations of cellulose, hemicellulose, lignin, and xylan [46] explains the abundance of *Xylanimonas, Xanthobacter*, *Xanthomona*s, and *Cellulomonas* found in these samples. These genera are associated with high-cellulose and xylan ecosystems, like decayed wood and bark [49], [50], [51].

In contrast to soil and bark, the micro-environments associated with leaves are generally considered nutrient-limited [52], [4]. Grape skins, especially in the early stages of ripening, also provide a limited amount of nutrients to sustain bacterial growth [53], [54], [55]. Furthermore, the surface of leaves and grapes contains compounds such as stilbenes (resveratrol and derivatives), which are involved in plant and fruit defences against microbial activity [56], [57]. In our study, the most abundant genera on leaves and grapes were *Pseudomonas* and *Sphingomonas*. These ubiquitous genera are known for their ability to grow under low-nutrient conditions [58], [59]. These results are in agreement with the findings of Leveau and Tech [17], that these two genera are among the most prevalent on vine leaves and grapes. Our results concerning the presence on grapes and leaves of members of genera commonly found in soil such as *Curtobacterium* and *Bacillus*, is also in agreement with that study. This observation and the fact that some T-RFs were shared by soil and all plant ecosystems, raised the hypothesis of a possible ecological link between telluric bacteria and epiphytic communities on the aerial parts of plants.

The colonisation of internal tissues by soil bacteria that thrive as endophytes and spread from the roots to different aerial plant parts through xylem vessels is well established [9]. While the endophytic continuum in grapevines is confirmed, the role of soil bacteria in epiphytic colonization of grapevine parts requires further elucidation. The physical proximity between soil and the various grapevine parts make this hypothesis very likely. In the most common grapevine training systems, the vine trunk and canopy are near the ground, facilitating the migration of micro-organisms from the soil to aerial parts of the plant through rain splash, high winds, insects, etc., [60], [61], [62], [63]. Mechanical soil management, like tillage, a common practice used to control weeds in vineyards, may also contribute to the migration of telluric micro-organisms to the aerial part of the plant. By breaking up soil aggregates, tillage often generates dust that may deposit on leaves, berries, and trunk, inoculating them with bacteria.

The importance of trunk bark as a potential source of inoculum for leaves and grapes should be also considered. They are physically very close to each other and may even come into direct contact. Prior investigations revealed the presence in bark of grape berry pathogenic fungi, such as *Uncinula necator* [62], [64], [22], *Botrytis cinerea, Fusarium laterium, Penicillium* spp., *Phomopsis viticola*, [65], [66], and the yeast-like fungus *Aureobasidium pullulans*, known for its biocontrol activity on grape berry pathogens. There is little information on the bacterial population in bark. However, Munkvold and Marois [65], found bacterial strains belonging to the *Pseudomonas* and *Bacillus* genera. Our study confirmed the presence of those genera on bark and grapes, suggesting that the bark community may influence leaf and grape-berry population structure.

The interaction between bacterial populations in vineyard soil and the epiphytic bacteria present on the various parts of grapevines, suggests that part of the plant epiphytic population may have a telluric origin. In addition, the bacterial population of the vegetative (leaf) and reproductive (fruit) structures of the vine may also be affected by trunk bark.

This first investigation is of particular importance, considering the role of bacteria in plant health and the fact that grape berries are the primary source of microbial communities that play a prominent role in the winemaking process and impact wine quality.

Further research is required to extend these observations and explore the ecological interaction between these different ecosystems. The use of other culture-independent approaches, like next-generation sequencing methods, will make it possible to present a complete survey of the bacterial communities on grape berries, leaves, and bark, and in vineyard soil.
